# Editorial: Thyroid hormones and cardiac arrhythmia

**DOI:** 10.3389/fendo.2022.1024476

**Published:** 2022-09-06

**Authors:** Johannes W. Dietrich, Patrick Müller, Melvin Khee Shing Leow

**Affiliations:** ^1^ Diabetes, Endocrinology and Metabolism Section, Department of Internal Medicine I, St. Josef Hospital, Ruhr University Bochum, Bochum, Germany; ^2^ Diabetes Centre Bochum/Hattingen, Klinik Blankenstein, Hattingen, Germany; ^3^ Centre for Rare Endocrine Diseases, Ruhr Centre for Rare Diseases (CeSER), Ruhr University Bochum and Witten/Herdecke University, Bochum, Germany; ^4^ Centre for Diabetes Technology, Catholic Hospitals Bochum, Ruhr University Bochum, Bochum, Germany; ^5^ Department for Electrophysiology, Medical Hospital I, Klinikum Vest, Recklinghausen, Germany; ^6^ Singapore Institute for Clinical Sciences (SICS), Agency for Science, Technology and Research (ASTAR), Singapore, Singapore; ^7^ Department of Endocrinology, Tan Tock Seng Hospital, Singapore, Singapore; ^8^ Metabolic Disorders Research Programme, Lee Kong Chian School of Medicine, Singapore, Singapore; ^9^ Cardiovascular and Metabolic Disorders Program, Duke-National University of Singapore Medical School, Singapore, Singapore

**Keywords:** thyroid function, cardiac arrhythmia, cardiovascular mortality, sudden cardiac death, MACE, hypothyroidism, thyrotoxicosis, cardiometabolic medicine

## The thyro-cardiac axis: Growing attention to a long-known connection

Among the premier effects of thyroid hormones in vertebrates are actions on the cardiovascular system (1–5). Cardiovascular complications rank among the key causes of death in thyroid emergencies (6).

**Figure 1:**
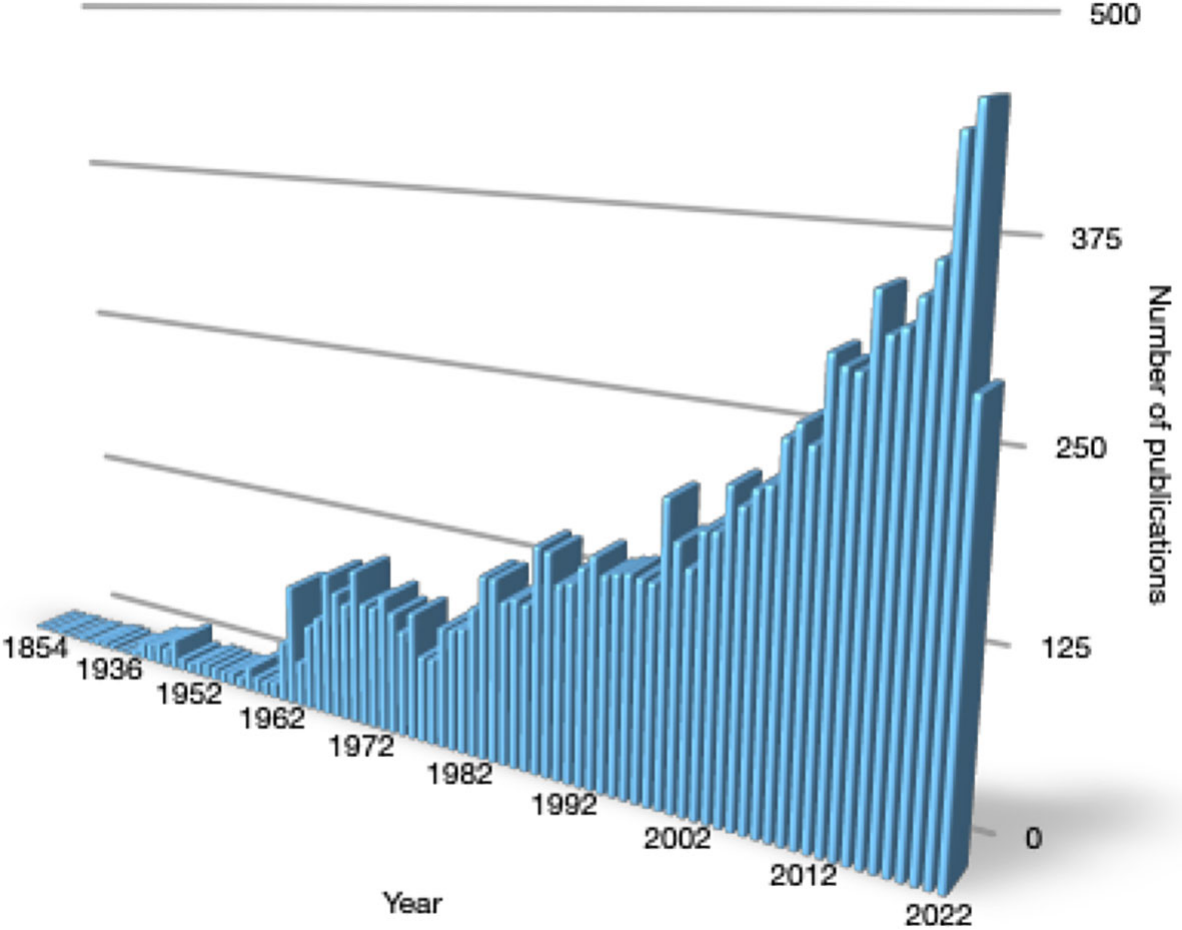
Yearly number of publications meeting the search term “thyroid AND heart” from 1854 to August 2022 in PubMed.

While our knowledge of the link between thyroid disease and cardiac arrhythmia dates back more than two centuries, attention to this connection continues to rise. The number of publications meeting the search formula “thyroid AND heart” is approaching the mark of 10,000 results (**Figure 1**), and it is still exponentially growing. A considerable proportion of these publications covers cardiac arrhythmia.

Therapeutic algorithms protocolized by cardiovascular medicine benefitted the prognosis of patients with chronic heart disease, and preventive programs have reduced cardiovascular morbidity at the population level. However, a substantial residual hazard persists. Therefore, the rediscovery of thyroid function as a major determinant of cardiovascular health is timely to address this gap of fundamental significance.

This Research Topic was initiated to provide a forum on recent developments in the thyro-cardiac nexus and to foster a deeper physiological understanding of this vitally important intersection. A series of articles summarising the state of current evidence deliver new perspectives on recent developments.

## Thyroid dysfunction and supraventricular arrhythmia

The resting heart rate is strongly modulated by thyroid hormone. This is the reason, why it became integral to established scoring systems for the diagnosis of myxoedema coma and thyroid storm (7–10). Thyrotoxicosis may also potentiate ectopic beats and atrial fibrillation (AF) (11–14). In adults, AF is a common condition conveying significant health hazard. Current guidelines recommend assessing the thyroid status in subjects with AF and, vice versa, screening for AF in hyperthyroid patients (15).

Subclinical hyperthyroidism and even FT4 concentration in its highest quartile in persons with normal TSH are associated with increased incidence of AF (16). Gencer et al. review this observation and provide recommendations for the management of new-onset thyroid-related AF.

Unlike in adults, AF is exceedingly rare in children and adolescents. Subramonian et al. describe the case of a 15-year-old female with AF and thyrotoxic tachymyopathy due to Graves’ disease, being reverted after successful treatment of the thyroid condition.

Subclinical hyperthyroidism is also associated with an increased risk for the recurrence of AF after catheter ablation, as demonstrated in the retrospective cohort study by Li et al.

## Thyroid function and ventricular arrhythmic events

In the ventricles, thyrotoxicosis promotes triggered activity and the formation of re-entry circuits giving rise to ventricular tachycardia, flutter and fibrillation (17, 18). These forms of malignant arrhythmia contribute to the high fatality rate of untreated thyroid storm.

This is exemplified by the case of a young woman with Graves’ disease and overt hyperthyroidism described by Fu et al. She suffered a cardiac arrest due to ventricular fibrillation but ultimately recovered following multimodal intensive care treatment including targeted temperature management with an intravascular cooling system.

## Allostatic load – confounder or mediator of risk?

The hypothalamus-pituitary-thyroid axis can dynamically adapt to physiological requirements. In type 1 allostatic load (e.g. in starvation or critical illness) the set point of the feedback loop is lowered, so that the concentrations of T3 (and occasionally TSH and T4) are down-regulated (19). A largely opposite endocrine pattern is observed in type 2 allostatic load (e. g. in psychosocial stress or certain psychiatric diseases) (19, 20).

Allostatic responses may raise considerable problems in the differential diagnosis of thyroid function. We (Dietrich et al.) demonstrated this for TSH, which correlates to a marker of type 2 allostatic load. Therefore, the prevalence of subclinical hypothyroidism may be overestimated in chronic psychosocial stress (21–24).

Low-T3 syndrome as the key component of thyroid allostasis in critical illness, tumours, uraemia and starvation (TACITUS) or non-thyroidal illness syndrome (NTIS) can be maladaptive and heralds a poor prognosis in severe illnesses. Two independent studies (Gao et al. and Abdu et al.) could demonstrate that this also applies to myocardial infarction with nonobstructive coronary arteries (MINOCA), a major subtype of type 2 myocardial infarction.

## Clinical evidence from prospective studies

Overt thyroid dysfunction is an established risk factor for mortality and major adverse cardiovascular events (MACE) (25–29). For minor disorders of thyroid function (e.g. subclinical hypo- and hyperthyroidism and within-reference range variations of thyroid hormones), the evidence was less clear. A systematic review including 32 studies covering more than 1.3 million subjects and a subsequent meta-analysis (Müller et al.) found cardiovascular death (CVD) to be predicted by both subclinical hypo- and hyperthyroidism. Circulating FT4 concentration was positively associated with the hazard ratio for CVD and MACE. The results suggest a monotonic association of FT4 to cardiovascular risk, but a complex U-shaped pattern linking TSH to cardiovascular endpoints, supporting the assumption of heterogeneous pathophysiological mechanisms.

## Physiological evidence and molecular mechanisms

In the clinical setting, the risk for malignant arrhythmia can be estimated e.g. by measuring certain time intervals in the electrocardiogram (ECG). In addition to the QT interval, an established biomarker of thyroid function (30), we could show that the Tp-e and JT intervals, which are not affected by QRS duration, correlate to TSH, FT4 and FT3 concentration (Aweimer et al.).

The impact of thyroid hormones on cardiac electrophysiology is mediated by a plethora of mechanisms (31). This applies to normal automaticity, triggered activity, disorders of impulse conduction and re-entry mechanisms. At a molecular level, thyroid hormones act *via* four distinct signalling types involving different time scales ranging from minutes to hours, where the expression of multiple genes is profoundly modulated (Müller et al).

## Aspects of therapy and prevention

Given the risk conferred by even slight deviations of thyroid hormones from an individual optimum, it is of paramount importance to restore euthyroidism as fast as possible in the case of thyroid emergencies (6). For thyroid storm, Lim et al. describe several options like pharmacotherapy and therapeutic plasma exchange, supported by adjuvant extra-corporal systems, including continuous renal replacement therapy (CRRT) and extracorporeal membrane oxygenation (ECMO). Irreversible brain damage in thyroid storm may be prevented by intravascular cooling devices, as demonstrated by Fu et al. For thyroid-related AF, Gencer et al. provide a comprehensive flow chart focussing on adjuvant non-endocrine treatment modalities.

Special caveats apply to substitution therapy with levothyroxine in hypothyroidism. In certain stages of thyroid cancer, TSH-suppressive therapy is recommended by current guidelines (32), but it may confer increased cardiovascular risk, as reviewed by Gluvic et al., thereby implying the need to evaluate its benefit:risk ratio and hence individualize the degree of TSH suppression that optimizes the health outcome.

## Prospectus

This collection of articles provides a current overview of the interface between thyroid function and cardiac rhythmology from different perspectives. According to the available evidence, even slight deviations of thyroid hormones confer significant risks for MACE, including malignant arrhythmia. Advanced diagnostical strategies should address the whole feedback loop to avoid misinterpretation by allostatic load, hysteresis and other effects, and modern therapeutic measures should involve multimodal approaches (21–23, 33–37). This is of particular importance due to the biological potency and relatively long plasma half-life of thyroid hormones.

The findings presented in this Research Topic also affect the ongoing debate about cardiometabolic medicine (38–40), strongly supporting the integration of thyroidology and cardiovascular rhythmology in this emerging subspecialty.

## Author contributions

JD, PM, and ML wrote some of the papers in this Research Topic and participated as guest editors for manuscripts, where they were not co-authors themselves. All authors listed have made a substantial, direct, and intellectual contribution to this editorial and approved it for publication.

## Acknowledgments

JD, PM and ML thank all authors, reviewers, and external editors for their valuable contributions to this Research Topic.

## Conflict of interest

The authors declare that the research was conducted in the absence of any commercial or financial relationships that could be construed as a potential conflict of interest.

## Publisher’s note

All claims expressed in this article are solely those of the authors and do not necessarily represent those of their affiliated organizations, or those of the publisher, the editors and the reviewers. Any product that may be evaluated in this article, or claim that may be made by its manufacturer, is not guaranteed or endorsed by the publisher.
